# Anti-inflammatory effects of hypoxia-preconditioned human periodontal ligament cell secretome in an experimental model of multiple sclerosis: a key role of IL-37

**DOI:** 10.1096/fj.201700524R

**Published:** 2017-08-23

**Authors:** Sabrina Giacoppo, Soundara Rajan Thangavelu, Francesca Diomede, Placido Bramanti, Pio Conti, Oriana Trubiani, Emanuela Mazzon

**Affiliations:** * Istituto di Ricovero e Cura a Carattere Scientifico (IRCCS) Centro Neurolesi Bonino-Pulejo, Contrada Casazza, Messina, Italy;; † Stem Cells and Regenerative Medicine Laboratory, Department of Medical, Oral, and Biotechnological Sciences, University G. d’Annunzio, Chieti, Italy;; ‡ Immunology Division, Postgraduate Medical School, University of Chieti-Pescara, Chieti, Italy

**Keywords:** inflammation, EAE, hPDLSC secretome, eperimental autoimmune ensephalomyelitis

## Abstract

Recent research has widely investigated the anti-inflammatory effects of mesenchymal stem cells and their secretory products, termed the secretome, in the treatment of multiple sclerosis (MS). The present study examined the capacity of the conditioned medium (CM) from human periodontal ligament stem cells (hPLSCs) under hypoxia (H-hPDLSCs-CM) to suppress experimental autoimmune encephalomyelitis (EAE), a murine model of MS. To induce EAE, female C57BL/6 mice were immunized with myelin oligodendroglial glycoprotein peptide_35–55_. At the onset of symptoms, H-hPDLSCs-CM was infused *via* the tail vein of mice. Our results demonstrate the efficacy of H-hPDLSCs-CM treatment in diminishing clinical and histologic disease score. A key finding from this study is the marked expression of anti-inflammatory cytokine IL-37, paralleled by the suppression of proinflammatory cytokines in mice with EAE that were treated with H-hPDLSCs-CM. In addition, a consequent modulation of oxidative stress, autophagic, and apoptotic markers was observed in mice with EAE after hPDLSCs-CM administration. In addition, to provide additional evidence of the molecular mechanisms that underlie H-hPDLSCs-CM, we investigated its therapeutic action in scratch injury–exposed NSC-34 neurons, an *in vitro* model of injury. This model reproduces severe inflammation and oxidative stress conditions as observed after EAE damage. *In vitro* results corroborate the ability of hPDLSCs-CM to modulate inflammatory, oxidative stress, and apoptotic pathways. Taken together, our findings suggest H-hPDLSCs-CM as a new pharmacologic opportunity for the management of MS.—Giacoppo, S., Thangavelu, S. R., Diomede, F., Bramanti, P., Conti, P., Trubiani, O., Mazzon, E. Anti-inflammatory effects of hypoxia-preconditioned human periodontal ligament cell secretome in an experimental model of multiple sclerosis: a key role of IL-37.

Multiple sclerosis (MS) is a chronic inflammatory, demyelinating disease of unknown trigger and complex pathology that causes myelin degradation and alteration of CNS functions (1, 2). Although some progress has been made in MS treatment in the past few years, conventional therapies provide only palliative relief and no definitive cure (3). Thus, the discovery of innovative approaches to improve outcomes for patients with MS is an objective of primary importance. In this context, a wide range of secreted factors that are produced by mesenchymal stem cells (MSCs), including growth factors, chemokines, and cytokines, which are generally defined as the MSC secretome, has shown great potential for the treatment of MS (4–6). In particular, neural crest MSCs from human dental tissues, such as periodontal ligament, dental pulp, and gingiva, received considerable interest because of the minimal invasive procedure involved in their collection, the remarkable differentiation ability to neurogenic and other cell lineages, economical cost, and the freedom from ethical concerns (7–9).

We recently demonstrated the immunosuppressive role of human periodontal ligament stem cell (hPDLSC)–derived conditioned medium (CM) and purified exosomes in experimental autoimmune encephalomyelitis (EAE), a murine model of MS (10). A large body of evidence suggests that paracrine mechanisms mediated by soluble factors that are released from MSCs play an important role in improving disease progression (11–13). Upon receiving pathologic signals, like inflammatory signals, these bioactive factors are homed to the site of injury, where they inhibit proinflammatory responses, prevent apoptosis, and induce tissue-specific precursor cell differentiation (14).

Of interest, some researchers have suggested the possibility of improving the capacity of the secretome to produce several of these factors *via* different culture methods (15). Among these, MSCs that are exposed to an hypoxic environment have been shown to greatly improve genetic stability and migration response to growth factors, chemokines, and inflammatory cytokines compared with MSCs under normoxic conditions (16, 17). Several studies have demonstrated the therapeutic properties of MSC-secreted factors that are stimulated by hypoxia in animal models of traumatic brain injury (18), massive hepatectomy (19), diabetic cardiomyopathy (20), and hindlimb ischemia (21); however, to date, there are no studies of its efficacy in MS treatment. Although the etiology of MS is not completely understood, there is no doubt about the efficacy of anticytokines in MS treatment. In this regard, recent studies have suggested an emerging role of IL-37, a member of the IL-1 family, as a new anti-inflammatory agent (22). IL-37 indeed plays a key role in the regulation of inflammatory response by lowering the levels of proinflammatory factors (23). To this end, we investigated, for the first time to our knowledge, whether CM from hPDLSCs under hypoxia (H-hPDLSCs-CM) could ameliorate EAE progression in an IL-37–dependent mechanism.

In addition, to provide additional evidence of the molecular mechanisms that underlie H-hPDLSCs-CM, we investigated its anti-inflammatory effects in an *in vitro* injury model of NSC-34 neurons induced by mechanical scratching. This *in vitro* model allows for the reproduction of the pathologic and physiologic changes of cells after trauma and, thus, may be useful for the identification of pharmacologic agents that exert effects directly on neurons that are subjected to injury (24).

## MATERIALS AND METHODS

### Ethics statement for human sampling

The procedure and informed agreement from human periodontal ligament biopsies were performed according to the approved guidelines of Medical Ethics Committee at the Medical School, G. d’Annunzio University (266/17.04.14). The formal consent form was signed by all participants before sample collection was carried out. The Department of Medical, Oral, and Biotechnological Sciences and the Laboratory of Stem Cells and Regenerative Medicine are certified in accordance with the quality standard ISO 9001:2008 (32031/15/S).

### hPDLSC culture establishment

Human periodontal ligament biopsies were collected from human premolar teeth that had been scheduled to be removed for orthodontic treatment. Samples were washed several times with PBS (LiStarFish, Milan, Italy) and cultured by using TheraPEAK MSC growth medium–CD (MSCGM-CD) BulletKit serum-free, chemically defined medium for the growth of human MSCs (Lonza, Basel, Switzerland) (25). Medium was changed twice a week, and cells that migrated from the explant tissue after reaching approximately 80% confluence were trypsinized (LiStarFish), then subcultured until passage 2.

For normoxic cultures, hPDLSCs were maintained at 95% air (20% O_2_), 5% CO_2_ in a normal incubator. Hypoxic culture conditions were generated as previously described by Ahmed *et al.* (26). H-hPDLSCs were maintained in a trigas incubator (AirTech, Tokyo, Japan). The culture chamber was formed from a plastic box that was connected to an outlet filter and a tube through which premixed gas—O_2_, CO_2_, and N_2_—was continuously injected. Humidified gas mixtures were composed of 3% O_2_, 6% CO_2_, and 91% N_2_ (Rivoira, Milan, Italy). Cells were put in the culture box to provide adequate humidification of cultures, then the culture box lid was closed.

### Preparation of H-hPDLSCs-CM

CM from H-hPDLSCs (15 × 10^3^ cells/cm^2^) that were cultured in xeno-free MSCGM-CD was collected after 24 h of incubation, then centrifuged at 1500 *g* for 15 min. Supernatant was collected, and 1 ml was subsequently resuspended in 3 ml of ice acetone and maintained overnight at 4°C, then centrifuged at 16,000 rpm for 12 min at 4°C (Centrifuge 5804 R; Eppendorf, Milan, Italy) (27). The pellet was lysed in RIPA and quantified by means Bradford assay.

### Animals

Female C57BL/6 mice (age 6–7 wk, weighing 20–25 g) were used for this experiment. Animals were acquired from Harlan (Milan, Italy) and housed in independently ventilated cages and maintained under 12-h light/dark cycles at 21 ± 1°C and 50–55% humidity with food and water *ad libitum*.

### Ethics statement for animal use

All animal care and use was performed according to the European Organization Guidelines for Animal Welfare. This study was authorized by the Ministry of Health General Direction of Animal Health and Veterinary Drug (A79E4.8–05/08/2016–D.lgs 26/2014). Experiments were planned in such a way to minimize the total number of mice that were necessary for the study.

### Induction of EAE

Mice were anesthetized with a combination of tiletamine and xylazine (10 ml/kg, i.p.). EAE was then induced in mice by using myelin oligodendrocyte glycoprotein peptide (MOG)_35–55_ (MEVGWYRSPFSRVVHLYRNGK; % peak area by HPLC ≥95; AnaSpec; EGT, Fremont, CA, USA) as reported by Paschalidis *et al.* (28). Mice were immunized with 300 μl/flank (s.c.) of emulsion that consisted of 300 μg MOG_35–55_ in PBS that was mixed with an equal volume of complete Freund’s adjuvant that contained 300 μg heat-killed *Mycobacterium tuberculosis* H37Ra (Difco Laboratories, Sparks, MD, USA). On the day of immunization and 48 h later, mice received an injection of 100 μl of *Bordetella pertussis* toxin (Sigma-Aldrich, Milan, Italy; 500 ng/100 μl, i.p.). The disease follows a course of progressive degeneration, with visible signs of pathology that consisted of flaccidity of the tail and loss of motion of the hind legs.

### Experimental design

Mice were randomly distributed into the following groups (*n* = 30 total animals): *1*) naive group *(n* = 5): mice did not receive an injection of MOG_35–55_ or other drugs and was used only as control. *2*) EAE group (*n* = 15): mice that were subjected to EAE with no other treatment. *3*) EAE + H-hPDLSCs-CM (*n* = 10): EAE mice were injected (tail) with H-hPDLSCs-CM (1.0 mg/mouse, i.v.) after 14 d from EAE induction.

Here, animals from the naive + H-hPDLSCs-CM group were not provided because, in our previous studies, we did not observe any effects as a result of H-hPDLSCs-CM injection. For this reason, as well as to minimize the number of mice used in our experiments, we have not included this group in our experimental design. On d 28 after EAE induction, all mice were euthanized with Tanax (Intervet Italia, Milan, Italy) (5 ml/kg body weight, i.p.). Spinal cord tissues were collected and processed for biochemical analyses. For this study, 2 independent experimental replicates *in vivo* were performed.

### Clinical disease score and body weight evaluation

On d 14 after EAE immunization, MS pathologic symptoms were observed, such as hindlimb paralysis, tail tonus reduction, and body weight loss. Severity of disease was evaluated by using the standardized 0–6 grading scale, as follows: 0, no signs; 1, partial flaccid tail; 2, complete flaccid tail; 3, hindlimb hypotonia; 4, partial hindlimb paralysis; 5, complete hindlimb paralysis; and 6, moribund or dead animal (29). Animals with a score of ≥5 were euthanized to avoid animal suffering. Body weight was also recorded daily from the day of immunization.

### Light microscopy

Spinal cord tissues [fixed in 10% (w/v) PBS-buffered formaldehyde] were embedded in paraffin and sectioned into 7-μm thin slices. After processing into xylene deparaffinization and subsequent rehydration steps, sections were stained with hematoxylin and eosin. Sections were visualized under optical microscope (ICC50HD; Leica Microsystems, Wetzlar, Germany).

### Immunofluorescence analysis of mouse spinal cord tissues

Paraffin-embedded tissue slices were deparaffinized with xylene, rehydrated with alcohol series, and incubated in 0.01 M citrate buffer (pH 6) for 30 min to retrieve antigen. Slices were then permeabilized for 10 min at room temperature by using Triton X-100 [0.1% (v/v)] in PBS and blocked with bovine serum albumin in PBS [3% (v/v)] for 20 min. Next, slices were incubated with selective primary Abs overnight at 4°C. Primary Abs applied for immunofluorescence analysis were as follows: anti–myelin basic protein (MBP) Ab [1:200 in PBS (v/v); Millipore, Billerica, MA, USA], anti-brain derived neurotrophic factor (BDNF) [1:50 in PBS (v/v); Santa Cruz Biotechnology, Santa Cruz, CA, USA], anti–cleaved caspase-3 [1:100 in PBS (v/v); Cell Signaling Technology, Danvers, MA, USA], anti-Bax [1:50 in PBS (v/v); Cell Signaling Technology], and anti–Bcl-2 [1:50 in PBS (v/v); Cell Signaling Technology]. Slices were then washed with Tween 0.1% in PBS and incubated with secondary Ab, Alexa Fluor 568 (Thermo Fisher Scientific, Waltham, MA, USA) for 1 h at 37°C. Samples were then incubated with TO-PRO for 1 h at 37°C to highlight cell nuclei.

Immunofluorescence images (*n* = 5 photos from each sample collected from all mice in each experimental group) were acquired by using confocal laser scanning microscopy (Zeiss LSM800 with Airyscan; Zeiss, Oberkochen, Germany). Images were acquired with a ×63 objective by using tiled area (150 μm × 150 μm) reconstruction method.

### Immunohistochemistry of mouse spinal cord tissue

Paraffin-embedded tissue slices were deparaffinized with xylene, rehydrated with alcohol series, and incubated in 0.01 M citrate buffer (pH 6) for 4 min to retrieve antigen. Slices were then incubated with 0.3% (v/v) hydrogen peroxide in 60% (v/v) methanol for 30 min to quench endogenous peroxidase and were blocked with normal goat serum in PBS [2% (v/v)] for 20 min. Next, slices were incubated with selective primary Abs overnight at 4°C. Primary Abs applied for immunohistochemical analysis are as follows: anti-CD4 [1:100 in PBS (v/v); Abcam, Cambridge, MA, USA) and anti-CD68 [1:200 in PBS (v/v); Biocare Medical, Concord, CA, USA]. Slices were then washed with PBS and incubated with avidin/biotin blocking reagent (DBA, Segrate, Italy) to block endogenous avidin and biotin binding sites. Next, slices were incubated with universal biotinylated secondary Ab followed by avidin horseradish peroxidase (HRP)–conjugated solution (Vectastain ABC Kit; Vector Laboratories, Burlingame, CA, USA) according to manufacturer’s instructions. Slices were then incubated with a hydrogen peroxide/DAB kit (Vector Laboratories) according to the manufacturer’s instructions. Counterstaining was performed with nuclear fast red (Vector Laboratories; pink background). To verify nonspecific background immunostaining, slices were incubated with either primary or secondary Ab alone. No staining was observed in these controls, which proved that the immunoreactions were positive in all experiments performed. Immunohistochemical images (*n* = 10 photos from each sample collected from all mice in each experimental group) were acquired by using light microscopy (Leica DM 2000 combined with LEICA ICC50 HD camera) and assessed by densitometric analysis by Leica Application Suite V4.2.0 software. Values are expressed as means ± sem of all mice for each group.

### Western blot analysis of mouse spinal cord tissues

Spinal cord tissues were homogenized by using ice-cold lysis buffer with the following ingredients: 10 mM Tris-HCl, pH 7.4, 0.32 M sucrose, 2 mM EDTA, 1 mM EGTA, 50 mM NaF, 5 mM NaN_3_, 10 mM 2-ME, and protease inhibitor tablets (Roche Applied Science, Monza, Italy). Homogenates were clarified by centrifugation at 1000 *g* for 10 min at 4°C, and the resulting supernatant was served as a cytoplasmic fraction. Pellets were further lysed by using ice-cold extraction buffer that consisted of 10 mM Tris-HCl, pH 7.4, 150 mM NaCl, 1 mM EDTA, 1 mM EGTA, 1% Triton X-100, and protease inhibitors. Homogenates were clarified by centrifugation at 15,000 *g* for 30 min at 4°C. The resulting supernatant was served as a nuclear fraction. Protein concentrations were assayed by using the Bio-Rad Protein Assay (Bio-Rad, Segrate, Italy). Proteins were subjected to SDS-PAGE, followed by blotting with PVDF membranes (Immobilon-P transfer membrane; Millipore). After blotting, PVDF membranes were stained with Ponceau S solution (Serva, Copenhagen, Denmark) to assess protein separation. Membranes were then incubated in blocking solution (5% skimmed milk in 1× PBS) for 45 min at room temperature. Membranes were subsequently incubated with selective primary Abs overnight at 4°C. Primary Abs used were as follows: IL-37 (1: 5000; R&D Systems, Minneapolis, MN, USA), caspase-1 (1:1000; Abcam), IFN-γ (1:250; Santa Cruz Biotechnology), JNK (1:250; Santa Cruz Biotechnology), iNOS (1:500; Cell Signaling Technology), COX-2 (1:500; Santa Cruz Biotechnology), beclin-1 (1:500; Cell Signaling Technology), LC3 (1:500; Cell Signaling Technology), phospho-PI3K (1:750; Cell Signaling Technology), PI3K (1:1000; Cell Signaling Technology), phospho-Akt (1:750; Cell Signaling Technology), Akt (1:1000; Cell Signaling Technology), phospho-mammalian target of rapamycin (mTOR; 1:750; Cell Signaling Technology), and mTOR [1:1000; Cell Signaling Technology) in 1× PBS, 5% (w/v) nonfat dried milk, 0.1% Tween-20]. Membranes were then incubated with HRP-conjugated goat anti-mouse IgG, HRP-conjugated goat anti-rabbit IgG, or HRP-conjugated anti-rat secondary Ab (1:2000; Santa Cruz Biotechnology) for 1 h at room temperature. To assess the equal loading of proteins, membranes were stripped and reprobed with HRP-conjugated glyceraldehyde 3-phosphate dehydrogenase Ab (1:1000; Cell Signaling Technology). Images of protein bands were visualized by using an ECL system (Luminata Western HRP Substrates; Millipore), and then acquired and quantified with ChemiDoc MP System (Bio-Rad) and a computer program (ImageJ; National Institutes of Health, Bethesda, MD, USA), respectively. Blots are representative of 3 separate and reproducible experiments. Data are expressed as means ± sem.

### Isolation of peripheral blood mononuclear cells and Western blot analysis on CD4^+^ T cells

Peripheral blood was collected into heparinized tubes, diluted to 1.5 ml final volume with PBS, and stored at 4°C for <4 h. Samples were layered onto Ficoll Paque Plus, and peripheral blood mononuclear cells were isolated by using a density gradient separation technique. An automated magnetic bead–based positive selection protocol was used to isolate CD4^+^ T cells (Stemcell Technologies, Vancouver, BC, Canada). All Western blot analyses were then performed according to standardized protocols above described. Here, primary Abs used were as follows: IL-17 (1:250; Santa Cruz Biotechnology) and IFN-γ (1:250; Santa Cruz Biotechnology). Representative blot images from 3 separate analyses are given.

### *In vitro* NSC-34 neuron cell culture

Murine-origin NSC-34 motoneuron cells were purchased from Cellutions Biosystems (Toronto, ON, Canada). High glucose-DMEM (Sigma-Aldrich) mixed with 10% fetal bovine serum (Sigma-Aldrich) was used to culture the neurons in a CO_2_ incubator under standard culture conditions as follows: 95% air/5% CO_2_ atmosphere; 37°C temperature. Cells with a passage number of <30 were used in the present study.

### *In vitro* injury model of NSC-34 neurons induced by mechanical scratching

Our *in vitro* injury model was produced by scratch injury as described earlier with minor modifications (30–32). In brief, NSC-34 neurons were cultured in 12-mm coverslips that were placed in 6-well culture plates by using 10% fetal bovine serum–supplemented DMEM. At 80% cell density, serum that contained DMEM was removed and cells were conditioned with serum-free DMEM for 12 h. To simulate mechanical damage, cells were subsequently subjected to injury by manual scratching (a sum of 4 scratches: 2 scratches vertically and 2 scratches horizontally) by using a 1-ml pipette tip, which produced a total of 9 quadrants. Space between the scratch areas was 2 mm. After injury, medium was removed to get rid of the floated cells, and cells were incubated with fresh serum-free medium for 24 h. Later, serum-free DMEM was discarded, and injured neurons were administered either fresh serum-free DMEM or DMEM that was mixed with H-hPDLSCs-CM (1 mg/ml), and were incubated for 24 h. Neuron cells without scratch injury were also included as control. Afterward, cells were either fixed or harvested for additional studies.

### Immunocytochemistry of NSC-34 neuron cells

To perform immunocytochemical staining, NSC-34 cells that were fixed with 4% paraformaldehyde were incubated with 3% hydrogen peroxide (Sigma-Aldrich) at room temperature for 15 min, followed by blocking with normal horse serum (Vector Laboratories) and Triton X-100 at room temperature for 20 min. Motoneurons were then incubated with the following primary Abs at 4°C overnight: iNOS (1:100; Cell Signaling Technology), COX-2 (1:50; Santa Cruz Biotechnology), TNF-α (1:50; Santa Cruz Biotechnology), IL-10 (1:50; Santa Cruz Biotechnology), Bcl-2 (1:50; Santa Cruz Biotechnology), Bax (1:50; Santa Cruz Biotechnology), cleaved caspase-3 (1:100; Cell Signaling Technology), BDNF (1:50; Santa Cruz Biotechnology), and neurotrophin-3 (NT3) (1:50; Santa Cruz Biotechnology). Cells were then exposed to biotinylated universal secondary Ab and streptavidin ABComplex-HRP per manufacturer instructions (Vectastain ABC Kit; Vector Laboratories). Immunostaining was developed with the DAB KIT (Vector Laboratories; immunoreaction produced a brown color). Counterstaining was performed with nuclear fast red staining (pink color).

Cells were analyzed by using a light microscope (Leica DM 2000 combined with a Leica ICC50 HD camera). Images (*n* = 3 images from each group) were obtained for densitometry evaluation to calculate the percentage of positive staining by using Leica Application Suite v.4.2.0 software. Data were acquired from 3 separate experiments performed in duplicate.

### Western blot analysis of NSC-34 neuron extracts

NSC-34 neuron extracts and proteins present in H-hPDLSCs-CM were quantified by using a Bio-Rad protein assay kit. Western blot procedures were performed as described for the spinal cord. Here, primary Abs used were as follows: TNF-α (1: 5000; Cell Signaling Technology), IL-10 (1:250; Santa Cruz Biotechnology), COX-2 (1:500; Santa Cruz Biotechnology), iNOS (1:500; Cell Signaling Technology), cleaved caspase-3 (1:500; Cell Signaling Technology), caspase 3 (1:500; Cell Signaling Technology), Bax (1:500; Cell Signaling Technology), Bcl-2 (1:500; Cell Signaling Technology), BDNF (1:250; Santa Cruz Biotechnology), nerve growth factor (1:250; Abcam), NT3 (1:250; Santa Cruz Biotechnology), and TGF-β (1:500; Abcam). Representative blot images from 3 separate analyses are given.

### Statistical evaluation

GraphPad Prism (v.6.0; GraphPad Software, La Jolla, CA, USA) was used for statistical analysis of the data. Results were statistically analyzed by using 1-way ANOVA and *post hoc* Bonferroni’s test for multiple comparisons. *P* ≤ 0.05 was considered significant. All values are indicated as means ± sem of *N* experiments.

## RESULTS

### H-hPDLSCs-CM improves CNS histopathology and clinical severity in EAE mice

**Figure 1.**
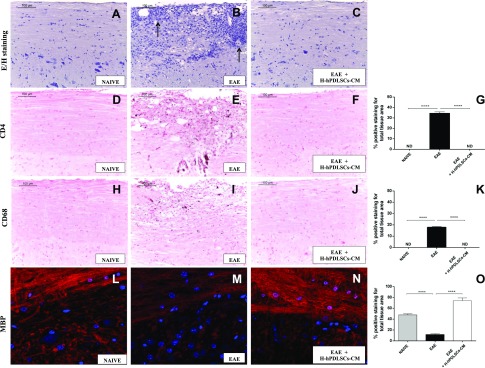
Hematoxylin and eosin staining. *A*, *B*) No histologic alteration was observed in spinal cord tissues from naive mice (*A*), whereas EAE mice (*B*) showed a wide area of infiltrating cells (arrows). *C*) H-hPDLSCs-CM treatment led to a complete resolution of inflammatory cell infiltration. *D*–*F*) Immunohistochemical analysis for CD4 in naive (*D*), EAE (*E*), and H-hPDLSCs-CM–treated mice (*F*). *G*) Densitometric analysis for CD4. *H*–*J*) Immunohistochemical analysis for CD68 in naive (*H*), EAE (*I*) and H-hPDLSCs-CM–treated mice (*J*). *K*) Densitometric analysis for CD68. *L*–*N*) Immunofluorescence analysis for MBP in naive (*L*), EAE (*M*), and H-hPDLSCs-CM–treated mice (*N*). *O*) Densitometric analysis for MBP. Naive *vs.* EAE and EAE *vs.* EAE + H-hPDLSCs-CM. ****P < 0.0001.

**Figure 2.**
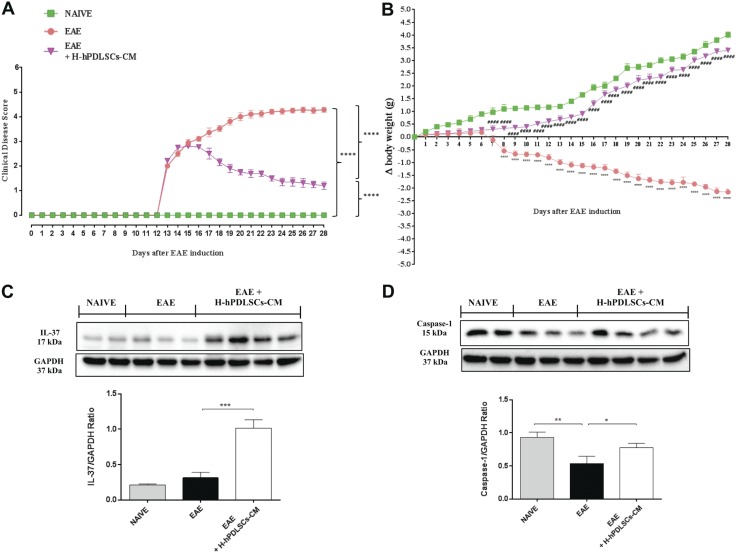
*A*) Clinical disease score. ****P < 0.0001, EAE *vs.* EAE + H-hPDLSCs-CM, and naive *vs.* EAE + H-hPDLSCs-CM. *B*) Body weight evaluations. ****P < 0.0001, naive *vs*. EAE; ^####^*P* < 0.0001, EAE *vs*. EAE + H-hPDLSCs-CM. Measurements of both clinical disease and body weight variations score are expressed as means ± SEM of all measurements of each experimental group. Results were analyzed by 1-way ANOVA, followed by Bonferroni’s test for multiple comparisons. *C*) Western blot analysis for IL-37. ***P = 0.0002, EAE *vs*. EAE + H-hPDLSCs-CM. *D*) Western blot analysis for caspase-1. *P = 0.0341 EAE *vs*. EAE + H-hPDLSCs-CM; **P = 0.0069, naive *vs*. EAE.

Hematoxylin and eosin staining revealed no histologic damage in naive mice (**Fig. 1*A***), whereas mice with EAE exhibited a marked infiltration of lymphocytes and polymorphonuclear cells in the white matter of the spinal cord (Fig. 1*B*, arrows). Treatment with H-hPDLSCs-CM led to a resolution of inflammatory cell infiltration (Fig. 1*C*). Immunohistochemical analysis revealed negative CD4 T-cell staining in spinal cord sections of naive animals (Fig. 1*D*), as well as in H-hPDLSCs-CM–treated mice (Fig. 1*F*). Conversely, untreated mice with EAE demonstrated positive staining for CD4 (Fig. 1*E*, see densitometric analysis; Fig. 1*G*). The degree of inflammation was also analyzed by investigating CD68 expression. Our results showed negative staining for CD68 in naive mice (Fig. 1*H*). In contrast, the increased macrophage expression observed in mice with EAE (Fig. 1*I*) was abolished by H-hPDLSCs-CM administration (Fig. 1*J*, see densitometric analysis; Fig. 1*K*). In the EAE model, demyelination occurred *via* myelin degradation, including the cleavage of MBP to smaller peptides for presentation to T cells, thereby sensitizing them to myelin damage. Thus, we investigated the expression of MBP, the main component of the myelin sheath. Immunofluorescence analysis for MBP demonstrated positive staining in naive mice (Fig. 1*L*), and negative staining in mice with EAE (Fig. 1*M*). The positive staining for MBP in EAE + H-hPDLSCs-CM mice instead revealed a marked remyelination after injection at the onset of EAE symptoms (Fig. 1*N*, see densitometric analysis; Fig. 1*O*). These results indicate that treatment with H-hPDLSCs-CM was able to reduce demyelination in the CNS of mice with EAE. Moreover, the histologic damage that was observed after EAE induction coincides with severe clinical score (**Fig. 2*A***) and loss of body weight (Fig. 2*B*), which were evaluated as parameters of disease. Mice with EAE exhibited a grading of disease with a mean clinical score of 4.28. Mice with EAE that were treated with H-hPDLSCs-CM instead showed significant improvement, with a clinical score as mean of 1.2, which was paralleled by the increase of body weight (+ 3.4 g). On the contrary, mice with EAE displayed significant body weight loss (−2.16 g). As expected, mice that belonged to the naive group exhibited a regular increase in body weight (+4.0 g) as well as the absence of clinical disease signs.

### IL-37 suppresses inflammatory response in mice with EAE that were treated with H-hPDLSCs-CM

**Figure 3.**
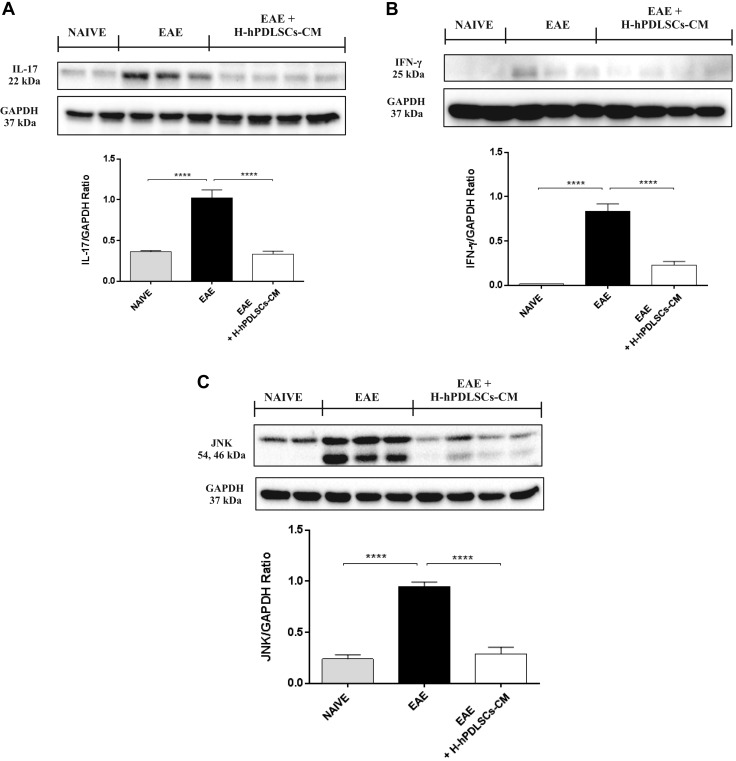
*A*) Western blot analysis for IL-17. *B*) Western blot analysis for IFN-γ. *C*) Western blot for JNK. ****P < 0.0001, naive *vs.* EAE and EAE *vs.* EAE + H-hPDLSCs-CM.

A key finding from this study is the marked expression of IL-37 in H-hPDLSCs-CM–treated mice. Western blot analysis displayed a basal nuclear expression of IL-37 in spinal cord samples from untreated mice with EAE, whereas treatment with H-hPDLSCs-CM significantly enhanced its expression (Fig. 2*C*). In addition, as IL-37 nuclear translocation is mediated by caspase-1 activation, we assessed its expression. Western blot analysis revealed an increased expression of caspase-1 in mice with EAE that were treated with H-hPDLSCs-CM compared with untreated mice (Fig. 2*D*). Once translocated into the nucleus, IL-37 suppresses the production of proinflammatory cytokines. In particular, during EAE progression, peripheral immune T cells [T helper (T_h_)] and glial cells are activated to produce a variety of proinflammatory cytokines. In this context, two diverse subsets of autoreactive T cells seem to be involved in MS pathogenesis, and, in particular, the IFN-γ–producing CD4^+^ T_h_1 and IL-17–producing T_h_17 cells. In view of this, we examined IL-17 and IFN-γ expression in CD4^+^ T cells. Western blot analysis demonstrated that both IL-17 and IFN-γ expression was significantly increased in CD4^+^ T cells from mice with EAE compared with the naive group. On the contrary, IL-17 and IFN-γ expression was reduced after treatment with H-hPDLSCs-CM (**Fig. 3*A, B***).

### IL-37 modulates the MAPK signal transduction pathway after H-hPDLSCs-CM treatment

Several studies have demonstrated that IL-37 inhibits the secretion of proinflammatory cytokines by modulating the expression of the MAPK pathway, which is generally involved in the regulation of many cell functions, including survival, proliferation, and apoptosis (33). Here, by investigating the expression of JNK, we observed that JNK expression is remarkably increased in spinal cord tissue taken from mice with EAE, whereas H-hPDLSCs-CM treatment attenuated its expression. A basal expression of JNK was detected in spinal cord samples from naive mice (Fig. 3*C*).

### Involvement of IL-37 in modulating oxidative stress markers in EAE mice treated with H-hPDLSCs-CM

**Figure 4.**
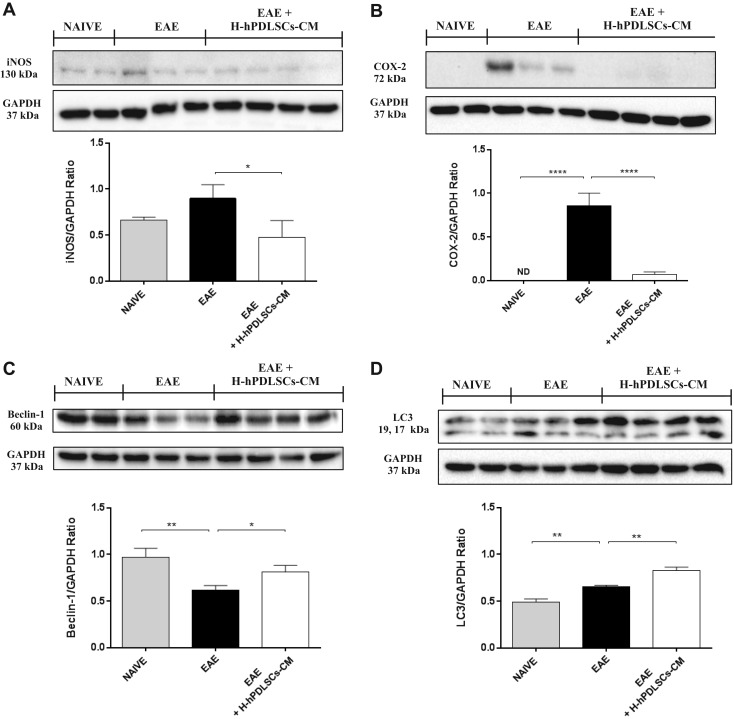
*A*) Western blot analysis for iNOS. EAE *vs.* EAE + H-hPDLSCs-CM. *P = 0.0349. *B*) Western blot analysis for COX-2. ****P < 0.0001, naive *vs*. EAE and EAE *vs*. EAE + H-hPDSSCs-CM. *C*) Western blot analysis for beclin-1. **P = 0.0041, naive *vs*. EAE; *P = 0.0321, EAE *vs*. EAE + H-hPDLSCs-CM. *D*) Western blot analysis for LC3. **P = 0.0041, naive *vs*. EAE; **P = 0.0019, EAE *vs*. EAE + H-hPDLSCs-CM.

As expected, one possible result of the upstream inhibition of the inflammatory pathway is the consequent blockage of the triggering of oxidative stress. We thus assessed the potential ability of IL-37 to prevent oxidative stress by evaluating the expression of iNOS and COX-2. Western blot analysis performed on spinal cord extracts revealed a basal expression level of iNOS and COX-2 in naive mice. In contrast, an enhanced expression for both markers was observed in spinal cord samples from mice with EAE that was reduced by administration of H-hPDLSCs-CM (**Fig. 4*A, B***).

### IL-37 regulates autophagy *via* the activation of the PI3K/Akt/mTOR pathway in EAE mice treated with H-hPDLSCs-CM

**Figure 5.**
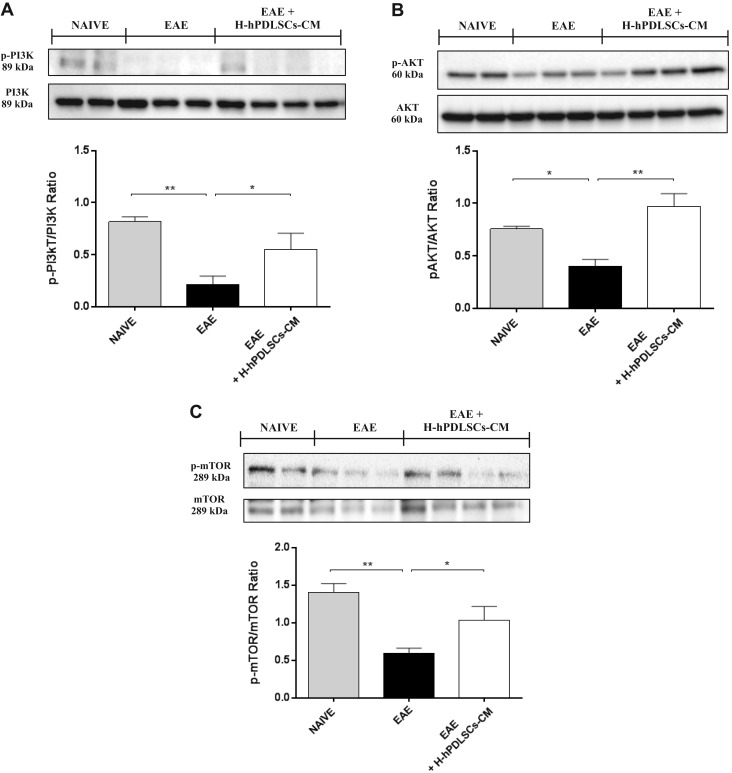
*A*) Western blot analysis for p-PI3K. **P = 0.0058, naive *vs*. EAE; *P = 0.0417, EAE *vs*. EAE + H-PDLSCs-CM. *B*) Western blot for p-AKT. *P = 0.0208, naive *vs*. EAE; **P = 0.0016, EAE *vs*. EAE + H-PDLSCs-CM. *C*) Western blot for p-mTOR. **P = 0.003, naive *vs*. EAE; *P = 0.0293, EAE *vs*. EAE + H-PDLSCs-CM.

The catabolic process for the autophagosomic-lysosomal degradation of proteins—autophagy—is activated mainly in response to nutrient deprivation, and it has also been associated with neurodegenerative diseases; however, the role of autophagy in MS is still unclear. Western blotting was performed to evaluate the expression of beclin-1 and LC3, which were chosen as principal markers of autophagy, in the spinal cord of mice with EAE. A down-regulation of beclin-1 and LC3 expression was observed in mice with EAE compared with naive mice. On the contrary, mice with EAE that were subjected to H-hPDLSCs-CM treatment demonstrated increased expression of beclin-1 as well as LC3 (Fig. 4*C*, *D*). Several reports suggest the possibility that IL-37 can regulate autophagy *via* an mTOR-dependent mechanism; however, the results are controversial. In view of this, we evaluated the role of the PI3K/Akt/mTOR pathway; specifically, we looked to the phosphorylation status of PI3K/Akt/mTOR, as its activation is mediated by phosphorylation of the proteins involved. Results demonstrated a substantial down-regulation of the PI3K/Akt/mTOR pathway in mice with EAE, as proven by the lower expression of p-PI3K (**Fig. 5*A***), p-AKT (Fig. 5*B*), and p-mTOR (Fig. 5*C*) in mice with EAE compared with naïve mice. Conversely, treatment with H-hPDLSCs-CM restored it, increasing significantly the phosphorylation of PI3K, Akt, and mTOR compared with the EAE group. No differences were observed between experimental groups for nonphosphorylated PI3K, Akt, and mTOR protein expression.

### Neurotrophic factor expression in EAE mice treated with H-hPDLSCs-CM

**Figure 6.**
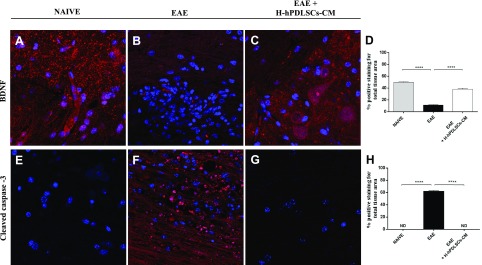
*A*–*C*) Immunofluorescence analysis for BDNF in naive (*A*), EAE (*B*), and EAE + H-PDLSCs-CM (*C*) mice. *D*) Densitometric analysis for BDNF. *E*–*G*) Immunofluorescence analysis for cleaved caspase-3 in naive (*E*), EAE (*F*), and EAE + H-PDLSCs-CM (*G*) mice. *H*) Densitometric analysis for cleaved caspase-3. ****P < 0.0001, naive *vs*. EAE and EAE *vs*. EAE + H-hPDLSCs-CM.

To investigate whether the therapeutic effects of H-hPDLSCs-CM could be a result of release of neurotrophic factors, we assessed the expression of BDNF, which is an important growth factor and is beneficial for neuronal function after neuronal damage. Thus, spinal cord sections were stained with an anti-BDNF Ab. Immunofluorescence data revealed positive immunolocalization for BDNF in naive mice (**Fig. 6*A***). Conversely, negative immunolocalization for BDNF was observed in untreated mice with EAE (Fig. 6B). When H-hPDLSCs-CM was administered, BDNF expression was restored at high levels, which preserved tissues that had been injured by EAE induction (Fig. 6*C*, see densitometric analysis; Fig. 6*D*).

### IL-37 regulates apoptosis in EAE mice treated with H-hPDLSCs-CM

**Figure 7.**
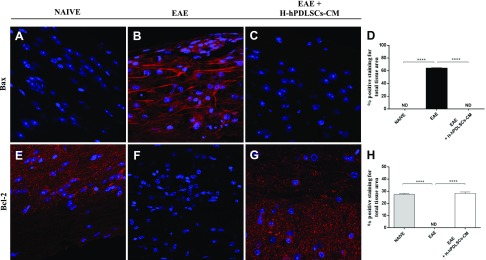
*A*–*C*) Immunofluorescence analysis for Bax in naive (*A*), EAE (*B*), and EAE + H-PDLSCs-CM (*C*) mice. *D*) Densitometric analysis for Bax. *E*–*G*) Immunofluorescence analysis for Bcl-2 in naive (*E*), EAE (*F*), and EAE + H-PDLSCs-CM (*G*) mice. *H*) Densitometric analysis for Bcl-2. ****P < 0.0001, naive *vs.* EAE and EAE *vs.* EAE + H-hPDLSCs-CM. ND, not detectable.

Finally, we investigated whether H-hPDLSCs-CM is able to protect against apoptosis that involves an IL-37–related mechanism. The mechanism of apoptosis is complex and involves a cascade of reactions; one of the key steps that leads to apoptosis is the leakage of cytochrome *C* from the mitochondria and activation of caspases. In the evaluation of the apoptotic pathway with immunofluorescence analysis, we observed completely negative staining for cleaved caspase-3 in spinal cord sections that were obtained from naive mice (Fig. 6*E*). In contrast, the EAE group exhibited an enhanced tissue expression for this marker (Fig. 6*F*). Treatment with H-hPDLSCs-CM reduced cleaved caspase-3 expression, which preserved tissues against EAE damage (Fig. 6*G*, see densitometric analysis; Fig. 6*H*). In addition, we observed completely negative staining for Bax and, in parallel, marked positive staining for Bcl-2 in naive mice (**Fig. 7*A, E***). On the contrary, after EAE induction, mice displayed positive staining for Bax and negative staining for Bcl-2 (Fig. 7*B, F*). Moreover, H-hPDLSCs-CM demonstrated a significant ability to protect the unbalance between Bax/Bcl-2 mice that were subjected to EAE (Fig. 7*C, G*; see densitometric analysis, Fig. 7*D, H*).

### H-hPDLSCs-CM reduces inflammation and oxidative stress in injured NSC-34 neurons

**Figure 8.**
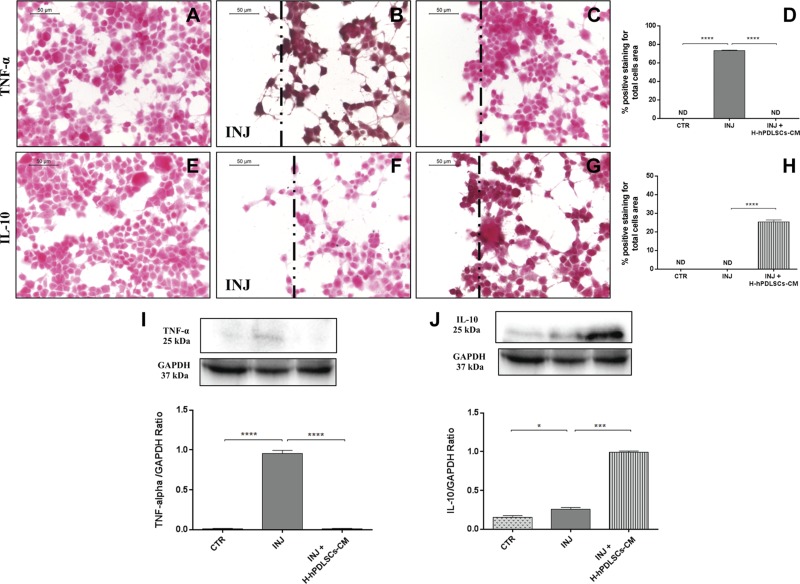
*A*–*C*) Immunohistochemistry analysis for TNF-α in control (CTR) cells (*A*), scratch-injured (INJ) neurons (*B*), and H-hPDLSCs-CM–treated scratch-injured neurons (*C*). *D*) Densitometric analysis for TNF-α. *E*–*G*) Immunohistochemistry analysis for IL-10 in CTR cells (*E*), scratch-injured neurons (*F*), and H-hPDLSCs-CM–treated scratch-injured neurons (*G*). *H*) Densitometric analysis for IL-10. Black dotted lines indicate the area of scratch injury. *I*) Western blot analysis for TNF-α. *J*) Western blot analysis for IL-10. *P = 0.0387, CTR *vs*. INJ; ***P = 0.0001, INJ *vs*. INJ + H-hPDLSCs-CM; ****P < 0.0001, CTR *vs*. INJ and INJ *vs*. INJ + H-hPDLSCs-CM. ND, not detectable.

**Figure 9.**
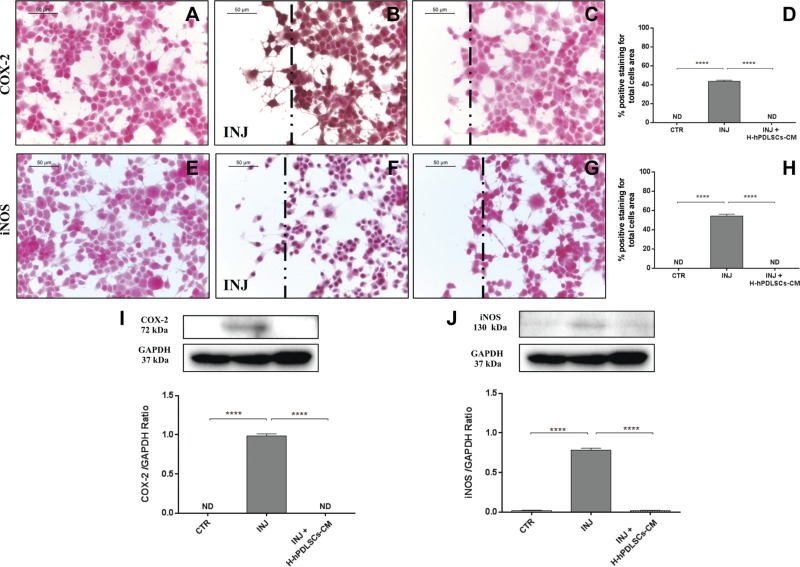
*A*–*C*) Immunohistochemistry analysis for COX-2 in control (CTR) cells (*A*), scratch-injured (INJ) neurons (*B*), and H-hPDLSCs-CM–treated scratch-injured neurons (*C*). *D*) Densitometric analysis for COX-2. *E*–*G*) Immunohistochemistry analysis for iNOS in CTR cells (*E*), scratch-injured neurons (*F*), and H-hPDLSCs-CM–treated scratch-injured neurons (*G*). *H*) Densitometric analysis for iNOS. Black dotted lines indicate the area of scratch injury. *I*) Western blot analysis for COX-2. CTR *vs.* INJ and INJ *vs.* INJ + H-hPDLSCs-CM. *J*) Western blot analysis for iNOS. ****P < 0.0001, CTR *vs.* INJ and INJ *vs.* INJ + H-hPDLSCs-CM. ND, not detectable.

To provide additional evidence for the molecular mechanisms that underlie the beneficial effects of H-hPDLSCs-CM, we performed *in vitro* studies on NSC-34 neuron cells. Scratch injury in NSC-34 neurons produced severe inflammation and oxidative stress. In control uninjured neurons, immunocytochemistry data demonstrated negative staining for TNF-α (**Fig. 8*A***), IL-10 (Fig. 8*E*), COX-2 (**Fig. 9*A***), and iNOS (Fig. 9*E*). Injured neurons displayed marked positive staining for proinflammatory cytokine, TNF-α (Fig. 8*B*), and oxidative stress markers, COX-2 (Fig. 9*B*) and iNOS (Fig. 9*F*). Conversely, treatment with H-hPDLSCs-CM completely reduced the activation of inflammation and oxidative stress. Negative staining was observed in injured neurons that were treated with H-hPDLSCs-CM for TNF-α (Fig. 8*C*), COX-2 (Fig. 9*C*), and iNOS (Fig 9*G*). Of interest, anti-inflammatory cytokine, IL-10, exerted significant positive staining in H-hPDLSCs-CM–treated injured neurons (Fig. 8*G*), whereas negative staining was observed for IL-10 in injured neurons without treatment (Fig. 8*F*). Densitometric quantification of TNF-α, IL-10, COX-2, and iNOS are shown in Figs. 8*D, H* and 9*D, H*. These results suggest that H-hPDLSCs-CM effectively suppresses activation of inflammation and oxidative stress in neurons that suffered scratch injury. Furthermore, modulation of TNF-α (Fig. 8*I*), IL-10 (Fig. 8*J*), COX-2 (Fig. 9*I*), and iNOS (Fig. 9*J*) after H-hPDLSCs-CM treatment was confirmed by Western blot analysis.

### H-hPDLSCs-CM inhibits apoptosis in injured NSC-34 neurons

**Figure 10.**
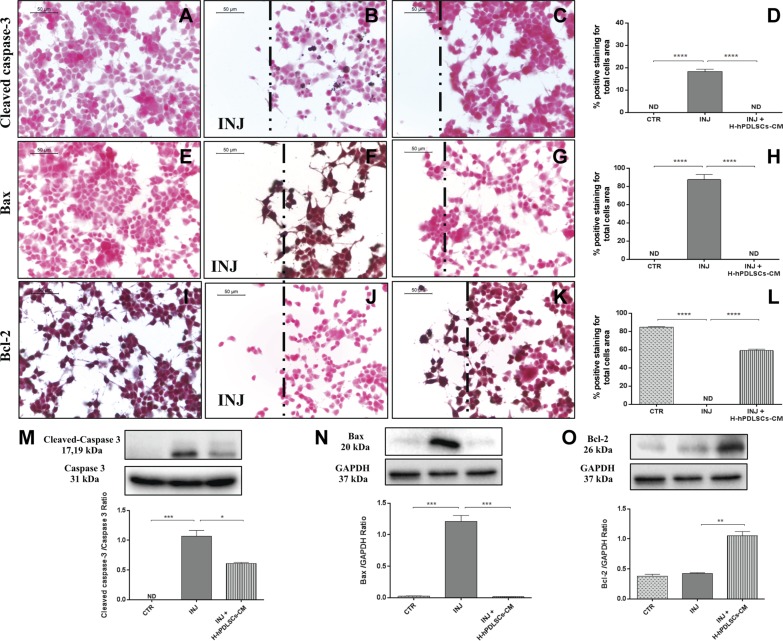
*A*–*C*) Immunohistochemistry analysis for cleaved caspase-3 in control (CTR) cells (*A*), scratch-injured (INJ) neurons (*B*), and H-hPDLSCs-CM–treated scratch-injured neurons (*C*). *D*) Densitometric analysis for cleaved caspase-3. *E*–*G*) Immunohistochemistry analysis for Bax in CTR cells (*E*), scratch-injured neurons (*F*), and H-hPDLSCs-CM–treated scratch-injured neurons (*G*). *H*) Densitometric analysis for Bax. *I*–*K*) Immunohistochemistry analysis for Bcl-2 CTR cells (*I*), scratch-injured neurons (*J*), and H-hPDLSCs-CM–treated scratch-injured neurons (*K*). *L*) Densitometric analysis for Bcl-2. Black dotted lines indicate the area of scratch injury. *M*) Western blot analysis for cleaved caspase-3. CTR *vs.* INJ; INJ *vs.* INJ + H-hPDLSCs-CM, *P = 0.0121. *N*) Western blot analysis for Bax. ****P < 0.0001, CTR *vs.* INJ and INJ *vs.* INJ + H-hPDLSCs-CM. *O*) Western blot analysis for Bcl-2. INJ *vs.* INJ + H-hPDLSCs-CM. **P = 0.0024. ND, not detectable.

We later investigated the activation of apoptosis induction in injured neurons. Immunohistochemistry data revealed that apoptosis was induced in neurons that had been injured by scratching. In injured neurons, proapoptotic proteins, cleaved caspase-3 (**Fig. 10*B***) and Bax (Fig. 10*F*), displayed significant positive staining. Moreover, negative staining was observed for the antiapoptotic protein, Bcl-2, in injured neurons without treatment (Fig. 10*J*). In contrast, H-hPDLSCs-CM totally suppressed the activation of apoptosis. In H-hPDLSC-CM–treated injured neurons, negative staining was observed for cleaved caspase-3 (Fig. 10*C*) and Bax (Fig. 10*G*). In addition, marked positive staining was observed for Bcl-2 in injured neurons that were treated with H-hPDLSCs-CM (Fig. 10*K*). In control uninjured neurons, negative staining was observed for cleaved caspase-3 (Fig. 10*A*) and Bax (Fig. 10*E*), and positive staining was observed for Bcl-2 (Fig. 10*I*). Densitometric quantification of cleaved caspase-3, Bax, and Bcl-2 is shown in Fig. 10*D, H, L*. In addition, the modulation of the apoptotic markers, cleaved caspase-3 (Fig. 10*M*), Bax (Fig. 10*N*), and Bcl-2 (Fig. 10*O*), after H-2hPDLSC-CM treatment was confirmed by Western blot analysis.

### H-hPDLSC-CM augments neurotrophic factors in injured NSC-34 neurons

**Figure 11.**
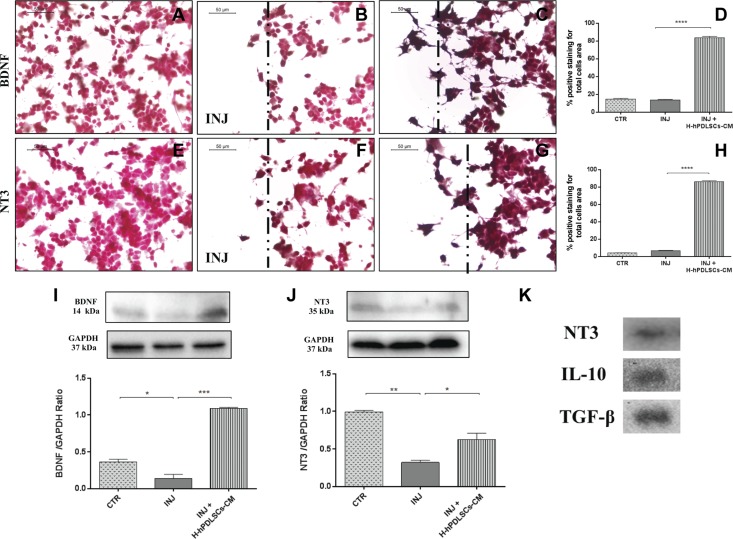
*A*–*C*) Immunohistochemistry analysis for BDNF in control (CTR) cells (*A*), scratch-injured (INJ) neurons (*B*), and H-hPDLSCs-CM–treated scratch-injured neurons (*C*). *D*) Densitometric analysis for BDNF. ****P < 0.0001, INJ *vs*. INJ + H-hPDLSCs-CM. *E*–*G*) Immunohistochemistry analysis for NT3 in CTR cells (*E*), scratch-injured neurons (*F*), and H-hPDLSCs-CM–treated scratch-injured neurons (*G*). *H*) Densitometric analysis for NT3. ****P < 0.0001, INJ *vs.* INJ + H-hPDLSCs-CM. Black dotted lines indicate the area of scratch injury. *I*) Western blot analysis for BDNF. *P = 0.0307, CTR *vs*. INJ; ***P = 0.0004, INJ *vs*. INJ + H-hPDLSCs-CM. *J*) Western blot analysis for NT3. **P = 0.0029, CTR *vs*. INJ; *P = 0.0287, INJ *vs*. INJ + H-hPDLSCs-CM. *K*) Western blot analysis for NT3, IL-10, and TGF-β in the extract of H-hPDLSCs-CM.****P < 0.0001. ND, not detectable.

We then investigated whether H-hPDLSC-CM could stimulate the expression of neurotrophic factors after injury. We found a basal level expression of the neurotrophic factors BDNF and NT3 in uninjured neurons (**Fig. 11*A, E***), as well as in injured neurons (Fig. 11*B, F*). In contrast, H-hPDLSC-CM treatment significantly augmented BDNF and NT3 expression in injured neurons (Fig. 11*C*, *G*). Densitometric quantification of BDNF and NT3 is shown in Fig. 11*D*, *H*. Finally, the increased expression of BDNF (Fig. 11*I*) and NT3 (Fig. 11*J*) in H-hPDLSC-CM–treated mice was confirmed by Western blot analysis. These findings suggest that H-hPDLSC-CM may provide neuroprotection by elevating the levels of BDNF and NT3.

### hPDLSC-CM contains trophic factor, NT3, and immunosuppressors, IL-10 and TGF-β

Lastly, we studied the presence of trophic factors and immunosuppressive cytokines present in H-hPDLSC-CM. Western blot data reveal the presence of NT3, IL-10, and TGF-β in H-hPDLSCs-CM (Fig. 11*K*), which suggests that the neuroprotective effect of H-hPDLSCs-CM in injured neurons might be, at least in part, a result of the presence of these growth factors and immunomodulatory cytokines. Ponceau-S staining of Western blot membrane of H-hPDLSC-CM is provided in Supplemental Fig. 1.

## DISCUSSION

The potential use of the MSC-derived secretome in the treatment of MS has received a great deal of interest in recent years (10, 34). In view of this, the secretome is a promising candidate for a novel cell-free therapy that allows us to overcome the limitations and risks of cell-based therapies, including immune incompetency, carcinogenicity, condition for *ex vivo* cell expansion, and cost (35). Of interest, it has been demonstrated that hypoxic preconditioning enhances the capacity of the secretome to produce a wide variety of chemokines, cytokines, and growth factors by which it may exert immunomodulatory and anti-inflammatory effects (15, 17, 18). In line with these findings, we demonstrated that treatment with H-hPDLSCs-CM effectively counteracts EAE progression and clinical impairment by reducing mainly the inflammatory pathway and the response to it. Moreover, understanding the mechanisms that regulate the production of soluble factors by MSCs is important and may result in the development of innovative treatments for MS. For this reason, we also investigated the effects of H-hPDLSCs-CM in scratch injury–exposed NSC-34 neurons, an *in vitro* model of injury that reproduces severe inflammation and oxidative stress conditions (24) that are commonly observed after EAE damage. After both MS and EAE onset, peripheral immune T cells and glial cells are activated to produce a variety of proinflammatory cytokines that exacerbate the inflammatory cascade (36); therefore, the discovery of anti-inflammatory mediators that are able to suppress inflammation and the immune response could have considerable importance in the MS treatment field. In this regard, recent studies have indicated the emerging role of IL-37, a member of the IL-1 family, as a new anti-inflammatory agent (37–39). Aberrant expression of IL-37 has been reported in autoimmune diseases, such systemic lupus erythematosus (40), rheumatoid arthritis (41), inflammatory bowel disease (42), and psoriasis (43); however, its role in MS has not yet been documented. Only a recent paper demonstrated an increased serum level of IL-37 in patients with MS, which suggests that this cytokine is a part of a feedback loop to control the underlying inflammation in MS pathogenesis (44). IL-37 generally acts *via* an intracellular mechanism that translocates to the nucleus (22, 45, 46). Here, by investigating IL-37 nuclear expression, we observed a significant down-regulation of its expression in spinal cord from mice with EAE, which was restored by H-hPDLSCs-CM treatment. As caspase-1 is required for nuclear translocation of intracellular IL-37 and for the secretion of mature IL-37, we also investigated caspase-1 expression in mice with EAE. Our results show an enhanced expression of caspase-1 in mice with EAE that were treated with H-hPDLSCs-CM. IL-37, once translocated into the nucleus, prevents the transcription of proinflammatory cytokines and chemokines, such as IL-1α, IL-6, TNF, and CXCL2 (45, 47, 48). In agreement with this, we observed decreased levels of the proinflammatory cytokines, INF-γ and IL-17, after H-hPDLSCs-CM administration (37). The ability of H-hPDLSCs-CM to modulate proinflammatory and anti-inflammatory cytokine balance was also corroborated in NSC-34 neurons that bad been injured by mechanical scratching. Injured neurons exhibited augmented expression of the proinflammatory cytokine, TNF-α, which was abolished in H-hPDLSCs-CM–treated injured NSC-34 neurons. In parallel, H-hPDLSCs-CM–treated cells demonstrated a notable increase in the production of the anti-inflammatory cytokines, IL-10 and TGF-β. *In vitro* studies on LPS-stimulated macrophages have suggested that treatment with IL-37 inhibits proinflammatory cytokine production by reducing the phosphorylation of MAPK proteins (49). In agreement with this, our results demonstrate an increased expression of JNK in spinal cord tissues from mice with EAE, which was significantly attenuated by H-hPDLSCs-CM administration.

Moreover, we found that the anti-inflammatory effects exerted by H-hPDLSCs-CM were reflected together with the consequent inhibition of the triggering of oxidative stress (50). Here, we examined the expression of iNOS and COX-2, which were chosen as the main markers of oxidative stress. As expected, we observed an increased expression of both markers in spinal cord samples from mice with EAE. In contrast, this increased expression was reduced by treatment with H-hPDLSCs-CM. Likewise, we noticed a total absence of iNOS and COX-2 expression in H-hPDLSCs-CM–treated injured neurons. An accumulation of evidence in recent years argues that reactive nitrogen species are among the major intracellular signal transducers that support pleiotropic responses that range from autophagy to death (51, 52). Dysregulation of autophagy is recognized as playing a central role in several neurodegenerative diseases (53, 54); however, its role in MS remains unclear (55). Although some early studies consider autophagy to be a mechanism of cell death, in recent years, has emerged in hypothesis as a useful mechanism for protecting cells from apoptosis (56). In addition, given the increasing evidence that autophagy has substantial effects on the immune system, it would not be surprising if this process was involved in the IL-37–mediated immunosuppression in MS. Here, to explore the involvement of autophagy, we examined the expression of two key autophagic proteins, beclin-1 and LC3, which have previously been detected in the spinal cord from mice with EAE (55). In agreement with this, we observed a down-regulation of beclin-1 and LC3 expression in mice with EAE, which was conversely enhanced after H-hPDLSCs-CM treatment. Although there is currently no evidence of a connection between IL-37 and autophagy, some clues in the literature suggest the ability of IL-37 in regulating autophagy *via* an mTOR-related mechanism (22, 57, 58). Specifically, mTOR plays a central role in triggering autophagy by acting as the key upstream effector of the PI3K/AKT pathway (59–61). The aberrant expression of PI3K/Akt/mTOR signaling has already been demonstrated in mice with EAE, but there are no data on the potential correlation with autophagy dysfunction. Thus, we investigated the involvement of the PI3K/Akt/mTOR pathway in EAE. Our results show a clear down-regulation of PI3K/Akt/mTOR signaling after EAE induction, which was restored by H-hPDLSCs-CM treatment. These observations led to the hypothesis that H-hPDLSCs-CM may regulate autophagy *via* activation of an mTOR-related mechanism upstream induced by IL-37. As PI3K/Akt/mTOR signaling is also involved in processes of oligodendrocyte survival and axon myelination in the EAE model (62–64), its restoration could lead to the amelioration of demyelination and neuronal injury. Our histologic evaluations of spinal cord from H-hPDLSCs-CM–treated mice revealed the presence of few areas of demyelination, sporadic inflammatory cell infiltrates, and a total absence of infiltrating CD4^+^ T and CD68^+^ cells. Furthermore, the expression level of MBP—the main protein of myelinated axons—was significantly increased in H-hPDLSCs-CM–treated mice compared with untreated mice with EAE. Our results also demonstrate that H-hPDLSCs-CM treatment improved clinical features, such as disability score and body weight loss, likely *via* the production of neurotrophic factors. Indeed, a significant up-regulation of BDNF was observed in mice with EAE that were treated with by H-hPDLSCs-CM. Likewise, our *in vitro* evaluations confirmed an increased presence of the neurotrophins BNDF and NT3 in H-hPDLSCs-CM–treated injured neurons.

Finally, we evaluated whether H-hPDLSCs-CM was able to protect mice with EAE from apoptosis that involved an IL-37–related mechanism. A recent study revealed that IL-37 could indeed regulate apoptosis by preserving the Bax/Bcl-2 balance (58). Our results show that H-hPDLSCs-CM treatment inhibited cell death in mice with EAE. In particular, our results demonstrate decreased activity of proapoptotic markers, cleaved caspase 3 and Bax, in mice with EAE after treatment with H-hPDLSCs-CM. In parallel, H-hPDLSCs-CM treatment significantly enhanced the expression of antiapoptotic protein Bcl-2. Likewise, neurons that had been subjected to scratch injury exhibited increased expression of both proapoptotic markers, cleaved caspase 3 and Bax, and reduced expression of Bcl-2. H-hPDLSCs-CM treatment instead restored the balance between pro- and antiapoptotic markers.

In summary, our findings suggest that the anti-inflammatory effects of H-hPDLSCs-CM in mice with EAE are largely attributable to the remarked presence of anti-inflammatory cytokine IL-37 and the consequent reduction of proinflammatory mediators. Although additional studies are needed to demonstrate the molecular mechanism by which IL-37 modulates the balance between pro- and anti-inflammatory cytokines, we believe that IL-37 could be a promising tool in MS management.

## Supplementary Material

Supplemental Data

## Abbreviations

BDNF: brain derived neurotrophic factor
CM: conditioned medium
EAE: experimental autoimmune encephalomyelitis
H-hPDLSCs-CM: conditioned medium from human periodonal ligament stem cells under hypoxia
hPDLSC: human periodontal ligament stem cell
HRP: horseradish peroxidase
MBP: myelin basic protein
MOG_35–55_: myelin oligodendroglial glycoprotein peptide
MS: multiple sclerosis
MSC: mesenchymal stem cell
mTOR: mammalian target of rapamycin
NT3: neurotrophin-3
T_h_: T helper
