# Supplementary material for: Anti-inflammatory effects of hypoxia-preconditioned human periodontal ligament cell secretome in an experimental model of multiple sclerosis: a key role of IL-37
Source: FASEB J. 2017 Aug 23;31(12):5592–608. doi: 10.1096/fj.201700524R (PMC5690382; doi:10.1096/fj.201700524R)
Supplement: Supplemental Data [file supp_31_12_5592__index.html]

Anti-inflammatory effects of hypoxia-preconditioned human periodontal ligament cell secretome in an experimental model of multiple sclerosis: a key role of IL-37 — Supplemental Data 

# Anti-inflammatory effects of hypoxia-preconditioned human periodontal ligament cell secretome in an experimental model of multiple sclerosis: a key role of IL-37

## Supplemental Data

- Supplemental Data
